# Reliability and validity of the Khmer version of the 10-item Connor-Davidson Resilience Scale (Kh-CD-RISC10) in Cambodian adolescents

**DOI:** 10.1186/s13104-016-2099-y

**Published:** 2016-06-08

**Authors:** Chanmettachampavieng Duong, Cameron P. Hurst

**Affiliations:** Department of Community Development, Faculty of Development Studies, Royal University of Phnom Penh, Phnom Penh, Cambodia; Doctor of Public Health program, Faculty of Public Health, Khon Kaen University, Khon Kaen, Thailand; Center of Excellence in Biostatistics, Faculty of Medicine, Chulalongkorn University, Bangkok, Thailand; Faculty of Public Health, Khon Kaen University, Khon Kaen, Thailand

**Keywords:** Resilience, 10-item CD-RISC, Cambodian adolescent, Reliability, Validity

## Abstract

**Background:**

Resilience has been characterized as a defensive factor against the refinement of mental health problems. This study adapted the Connor–Davidson Resilience Scale (Kh-CD-RISC10) for use in Khmer adolescents and subsequently investigates its psychometric properties.

**Methods:**

Using stratified random sampling, this cross-sectional study sampled Cambodian adolescents from high schools selected randomly within three provinces (Phnom Penh, Battambang and Mondulkiri)—location (rural, urban) combinations. Parallel analysis was used to identify the number of component(s), and the structure of the single factor was subsequently explored using principal axis factoring. A confirmatory factor analysis was then performed to establish the fit of the Kh-CD-RISC10 to another sample. To assess convergent validity, the factor scores of the Khmer version of Connor–Davidson Resilience Scale were categorized into three levels, and then the general negative affectivity (GNA) and physiological hyperarousal (PH) scales (derived from the DASS 21) were compared among the three resilience groups.

**Results:**

Of the 798 participants who responded (responded rate = 82.26 %), 440 (41.23 %) were female and the age ranged from 14 to 24 years old (mean = 17.36, SD = 1.325). The internal consistency of the Khmer 10-item CD-RISC was also shown to be high in Cambodian adolescents (Cronbach’s alpha = 0. 82). Confirmatory factor analysis revealed the single factor model fit data adequately (χ^2^ = 100.103, df = 35, p < 0.001, CFI = 0.9484, RMSEA = 0.0384). We found that there were significant differences in both General Negative affectivity and Physiological Hyperarousal among the three resilience groups (F_GNA_ = 12. 84, df = 2, p < 0.001; F_PH_ = 13. 01, df = 2, p < 0.001).

**Conclusion:**

The results from the present study indicate that the Khmer version of CD-RISC shows good psychometric properties in Cambodian adolescents. Our result confirms that a single dimension underlay the 10 items on the CD-RISC scale of this population, and can be used to assess the resilience comparing to the level of PTSD symptoms in general Khmer adolescent.

## Background

Resilience has been characterized as a defensive factor against the refinement of mental health problems [1–5]. The definition of resilience can vary depending on the different stages of life and experience [6]. A further definition is given by Windle [7] who describes resilience as a procedure for conquering the negative impact, or adapting effectively to traumatic encounters, and evading the negative trajectories connected with risk. Other studies define resilience as being based on personal [8] or family characteristics [9]. Many studies consider the measurement of the construct of resilience, and several of these have considered measurement of this construct in adolescents [10–12].

In recent decades, Cambodia has experienced extreme societal breakdowns, accompanied by genocide and more common war. Violence and poverty have disabled Cambodia’s capacity to recuperate from the destruction initiated. Regardless of a developing economy and fast advancement, mental health specialists say the mental scars are yet to recuperate [13, 14], and there is likely to be a high prevalence of poor mental health among the Khmer people [15, 16]. For instance, several studies have demonstrated a high prevalence of post-traumatic stress disorder (PTSD) and depression [16–18]. However, other studies have shown that not all individuals who encounter trauma develop mental health problems, and resilience is a major moderator of response to trauma [2, 5].

Resilience has received little attention in Cambodia. Indeed, no validated instrument has been developed to measure resilience in either the Khmer population in general, or Khmer adolescents, in particular. This study adapted the Connor–Davidson Resilience items (Kh-CD-RIS10) for use in Khmer adolescents and subsequently investigates its psychometric properties.

## Methods

### Study design and population

A stratified random sample of Cambodian adolescents was collected from high schools within six province (Phnom Penh, Battambang and Mondulkiri)—location (rural, urban) combinations. We believe that urban and rural adolescents are at variance in ways that include both the physical and social environments, ranging from factors such as ability to access to the education system, employment, transport, healthcare and leisure facilities to noise, crowding, rates of crime and fear of crime [19]. People who live in rural areas experience better mental health than those whom are living in urban to an extent that was numerically modest but statistically significant. There are small but statistically significant differences in rates of common mental disorders between urban and rural residents [20]. A single class from each grade (10, 11 and 12) was selected (based on the availability) within each school, and all students were asked to participate. Finally, data from 798 students were obtained from the 970 questionnaires distributed (response rate = 82.26 %).

The study protocol was approved by the Khon Kaen University Ethics Committee for Human Research (HE572118; Thailand), National Institute of Public Health (0184 NECHR) and Ministry of Education, Youth and Sport (Cambodia). All participants provided informed consent or assent (for subject below 18 years old). Participation in the study of those under 18 years old also required parental informed consent.

### Measurement

The data collected in this study is a part of a larger study to investigate the level of posttraumatic stress disorder symptoms in Cambodian adolescents. All participants were asked to complete a series of questions relating to resilience, depression, anxiety and stress, posttraumatic stress disorder symptoms, and quality of life. In addition, socio-demographic variables were also collected including: gender, year of birth, school grade (10, 11 and 12), religion (Buddhism, Islam and Christianity), place of upbringing (rural and urban) and perception of family economic status (lower, medium, and upper).

#### Connor–Davidson Resilience Scale (CD-RIS-10)

The Connor–Davidson Resilience Scale (CD-RISC) is a widely used instrument for measuring resilience. The CD-RISC has 10 self-rated items, and has been identified as best psychometric scale to measure resilience [7]. This measurement is rated on a 5-point scale from 0 (not true at all) to 4 (true nearly all the time) and after combining into a single scale, a higher score indicates a higher level of resilience [21]. In order to administer to Cambodian adolescents, the questionnaire was forward and back translated from English to the Khmer language and minimal changes were required for the original Khmer translation.

#### Depression, anxiety and stress scales (DASS-21)

The depression, anxiety and stress scales (DASS-21) were used in our study to examine the convergent validity of the Khmer version of CD-RISC10. The DASS-21 is a well-established 21 item instrument to measure the negative emotional states of depression, anxiety, and stress [22–25]. Several studies have shown adolescents tend to not distinguish between depression, anxiety and stress, and instead DASS can only be used to gauge constructs such as general negative affectivity [23, 25], low positive affectivity and physical hyperarousal [26]. We found this to also be the case for Cambodian adolescents (results not shown).

### Statistical analysis

Participants were randomized into two groups (1:2 ratio) to explore (1/3 of participants) and confirm (2/3 of participants) the resilience measurement model. For exploratory factor analysis, parallel analysis (based on principal component analysis) was used to identify the number of component(s), and the structure of the factor(s) was subsequently explored using principal axis factoring.

Based on the results of the principal axis factoring, a confirmatory factor analysis was performed on the remaining held out participants. We deemed a successful model was that with a GFI > 0.9 [27] and a RMSEA < 0.08 [28]. The Adjusted Good-of-fit index (AGFI) was also reported, along with the χ^2^ statistic (a poor measure of model fit of measurement models, but included here for reasons of convention).

To assess convergent validity, the factor scores of the Khmer version of Connor–Davidson Resilience Scale were categorized into three levels: high, moderate and low resilience, and then both the general negative affectivity (GNA) and physiological hyperarousal (PH) scales (derived from the DASS 21) were compared among the three resilience groups. The low resilience group were represented by the first quartile (Kh-CD-RISC10 < 3.31), the moderate group were the middle 50 % (3.32–4.63) and high resilience group by the upper quartile (Kh-CD-RISC10 ≥ 4.64). Comparisons among resilience groups were then conducted using ANOVA, and Cohen’s d was used to gauge effect size.

Cronbach’s alpha coefficient was used to examine the internal consistency of the Khmer version of Connor–Davidson Resilience Scale. All analyses were conducted with R statistical language (version 3.1.1) [29]; parallel analysis was conducted using nFactor (version 2.3.3.) [30], principal axis factoring using psych (version 1.4.8.) [31], and confirmatory factor analysis using the sem (version 3.1-5) [32] R libraries. Statistical significance was set at *α* = 0.05 (two-tailed) for inferential analysis.

## Results

### Sample characteristics

Of the 798 participants who responded (response rate: 82.26 %), 440 (41.23 %) were female and the age ranged from 14 to 24 years old (mean = 17.36, SD = 1.325). Descriptive statistics of the 798 participants are provided in Table 1.

**Table 1:** Demographic characteristics of Khmer high school students

Characteristics	n = 798
Age (year)	
Mean (SD)	17.36 (1.32)
Range	14–24
School grade n (%)	
Grade 10	301 (37.72)
Grade 11	220 (65.29)
Grade 12	271 (34.04)
Gender n (%)	
Male	329 (42.78)
Female	440 (57.22)
Religion n (%)	
Buddhism	739 (94.26)
Islam	22 (2.81)
Christian	23 (2.93)
Place of upbringing n (%)	
Rural	345 (47.72)
Urban	378 (52.28)
Perception of family economic status n (%)	
Lower	98 (13.64)
Medium	395 (55.01)
Upper	225 (31.33)

*N* number, *SD* standard deviation, % percentage

### Construct validity

**Fig. 1:**
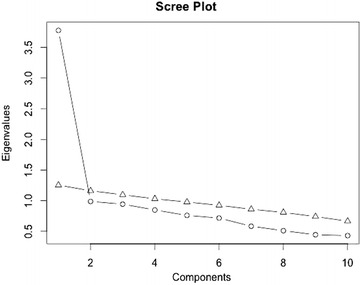
Parallel analysis of factor components of the Kh CD-RSIC 10. *Circle* represent the observed eigenvalue and *triangle* the expected eigenvalue under randomization

**Fig. 2:**
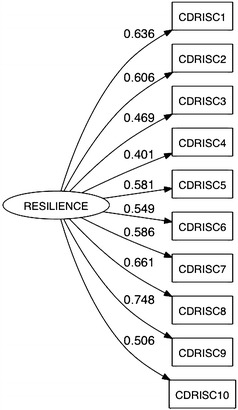
Standardized factor loading from the confirmatory factor analysis for the Khmer version of Connor–Davidson Resilience Scale (Kh-CD-RISC10) in Khmer adolescents. Total sample: n = 798, χ^2^ = 100.103, df = 35, p < 0.001, CFI = 0.9484, and RMSEA = 0.0384

After randomizing the data into two sets (1/3 of participants for exploratory factor analysis, and 2/3 for confirmatory factor analysis), parallel analysis was conducted and confirmed a single factor structure was appropriate (Fig. 1). Principal axis factoring was then employed to explore the factor structure and the resulting loadings are given in Table 2. Confirmatory factor analysis revealed the single factor model fit the data adequately (χ^2^ = 100.103, df = 35, p < 0.001, CFI = 0.9484, RMSEA = 0.0384). The loadings from the confirmatory factor analysis are provided in Fig. 2. The KMO was 0.82 and Bartlett’s sphericity test was significant (χ^2^ = 1297. 003, df = 45, p < 0.001) suggesting the factor solution was adequate.

**Table 2:** Factor loading of the Kh-CD-RSIC10 from principal axis factoring

Item	Description	Factor loading
1	I am able to deal with change	0.636
2	I can deal with whatever comes my way	0.605
3	I try to see the funny side of things when I am faced with problems	0.469
4	Dealing with stress can make me stronger	0.401
5	I tend to bounce back after being sick, injury, or other hardships	0.580
6	I believe I can achieve what I want, even there are problems	0.549
7	Under pressure, I still think clearly	0.585
8	I do not lose hope from failure	0.661
9	I think of myself as a strong person when dealing with life’s challenges and difficulties	0.747
10	I am able to handle unpleasant or painful feelings like sadness, fear and anger	0.506

We suspected that the nature of the KH-CD-RISC10 may differ between males and females, so a group analysis was subsequently performed. However, we found that there were no substantial differences between groups (ΔCFI < 0.01).

### Convergent validity

Using scores derived from the general negative affectivity (GNA) and physiological hyperarousal (PH) scales (from the DASS-21), we explored whether these factors were associated with resilience level (low, moderate, high) of the Kh-CD-RISC10. We found that there were significant differences in both GNA and PH among the resilience groups (F_GNA_ = 12.84, df = 2, p < 0.001; F_PH_ = 13.01, df = 2, p < 0.001). Table 3 gives the estimated means for GNA and PH for the three resilience groups, and effect sizes for the comparisons. Perusal of the group means and effect sizes (Table 3) shows that discrimination is highest among the high group compared to moderate and low groups.

**Table 3:** Mean score of the Khmer version of Connor–Davidson Resilience Scale component index by resilience category for general negative affectivity (GNA) and physiological hyperarosal (PH)

	Resilience						
	Low Resilience (1)	Moderate Resilience (2)	High Resilience (3)	p value	Effect size		
					1–2	1–3	2–3
GNA	3.61 (1.02)	3.58 (0.96)	3.25 (1.01)	0.0161	0.030	0.364	0.334
	n = 83	n = 165	n = 83				
PH	3.13 (0.97)	3.16 (0.93)	2.89 (0.79)	0.0089	0.033	0.264	0.297
	n = 83	n = 165	n = 83				

### Reliability

Based on the single factor model, the reliability of the Kh 10-item CD-RISC was evaluated for internal consistency (Cronbach’s alpha). The alpha value of 0.82 indicates sufficiently high reliability to provide confidence in interpreting the score. The correlation item total score ranged from 0.79 to 0.85.

## Discussion

The results of this study indicate that the Khmer version of Connor–Davidson Resilience Scale (Kh-CD-RISC-10) has good psychometric properties and is valid for use in late Cambodians adolescents. Our results confirm that a single dimension underlay the 10 items on the CD-RISC scale for this population. The Khmer version of Connor–Davidson Resilience Scale (CD-RISC) presented good internal consistency (*α* = 0.82, range of factor score = 0.401–0.748) which was comparable to the original version (*α* = 0.85) [33] and that used for an adolescent sample (*α* = 0.85, range of factor score = 0.48–0.76) [10]. We also found evidence that the Kh-CD-RISC10 converged with GNA and PH, especially discriminating those on the upper end of the resilience scale.

To date, there have been no studies that have evaluated the psychometric properties of a resilience measure in the Khmer population. The findings of this study are likely to be useful for mental health researchers considering the Cambodian population. Given the social upheaval in Cambodia’s recent history, and the high prevalence of poor mental health that is likely to have resulted from this, there is a strong need for validated measures of resilience and other mental health indicators in this population.

## Limitation

The sample of this study is comprised of high school students from grade 10, 11 and 12, therefore the result of this study may not be generalizable to younger adolescent and children or, the adult population. Another limitation of this study is the lack of evidence of the stability of the measurement across time (test–retest), because the questionnaire was only administered at one time point. However, this is the first validation study of the Khmer version of Connor–Davidson Resilience Scale (Kh CD-RISC) in Cambodia, or indeed, any measure of resilience for this population.
